# Hypoxia inducible factor-1 (HIF-1α) reduced inflammation in spinal cord injury via miR-380-3p/ NLRP3 by Circ 0001723

**DOI:** 10.1186/s40659-020-00302-6

**Published:** 2020-08-20

**Authors:** Xigong Li, Xianfeng Lou, Sanzhong Xu, Junhua Du, Junsong Wu

**Affiliations:** Department of Orthopaedic Surgery, The First Affiliated Hospital of Zhejiang University, No.79 Qingchun Road, Hangzhou, 310003 China

**Keywords:** HIF-1α, Circ 0001723, miR-380-3p, NLRP3, Spinal cord injury, Inflammation

## Abstract

**Background:**

Spinal cord injury (SCI) is a severe central nervous system trauma. The present study aimed to evaluate the effect of HIF-1α on inflammation in spinal cord injury (SCI) to uncover the molecular mechanisms of anti-inflammation.

**Results:**

HIF-1α was reduced in SCI model rats and HIF-1α activation reduced TNF-α, IL-1β, IL-6 and IL-18 levels in SCI model rats. Meanwhile, Circ 0001723 expression was down-regulated and miR-380-3p expression was up-regulated in SCI model rats. In vitro model, down-regulation of Circ 0001723 promoted TNF-α, IL-1β, IL-6 and IL-18 levels, compared with control negative group. However, over-expression of Circ 0001723 reduced TNF-α, IL-1β, IL-6 and IL-18 levels in vitro model. Down-regulation of Circ 0001723 suppressed HIF-1α protein expressions and induced NLRP3 and Caspase-1 protein expressions in vitro model by up-regulation of miR-380-3p. Next, inactivation of HIF-1α reduced the pro-inflammation effects of Circ 0001723 in vitro model. Then, si-NLRP3 also inhibited the pro-inflammation effects of Circ 0001723 in vitro model via promotion of autophagy.

**Conclusions:**

We concluded that HIF-1α reduced inflammation in spinal cord injury via miR-380-3p/ NLRP3 by Circ 0001723.

## Background

Spinal cord injury (SCI) can be divided into primary injury and secondary injury according to its pathogenesis [1]. Of them, primary injury is resulted from direct or indirect action of the initial force on the spinal cord [2]. In contrast, secondary injury is induced by a series of physiological and biochemical mechanisms on the basis of primary injury, such as oxidative stress, inflammatory response and excessive release of excitatory amino acid (EAA) [2]. These have resulted in self-destructive lesions in the intact lesion-surrounding tissues. Thus, they have further deepened the injury degree and expanded the injury range [3]. Post-SCI inflammatory reaction is relatively complicated, which involves the dynamic effects of all factors in the nervous and immune systems [3]. Therefore, it has become the important event constituting secondary injury [4]. Plenty of studies have suggested that SCI-induced inflammatory response has dual effect of neural damage and neural protection [3]. Consequently, its negative and positive effects on SCI repair should be utilized [5]. In this way, its repair potential can be selectively exerted while minimizing its destruction [5].

Circular RNAs (circRNAs), one kind of non-coding RNA, have been reported as numerous biological processes, such as inflammation, proliferation, apoptosis, and cell cycle regulation [6]. miRNA is an important regulatory molecule, which has become a new target of therapeutic intervention for promoting repair and regeneration [7]. In recent years, autophagy has gradually become the hotspot in SCI research. However, the role of early post-SCI autophagy activation remains a source of controversy [8]. In spinal cord injury, the expression of hypoxia regulatory protein HIF-1α is up-regulated, while the apoptosis-related protein c-parp is down-regulated [9]. Meanwhile, the autophagy-related protein LC3 is up-regulated [9]. Therefore, it is speculated that the hypoxia-induced decline in radiosensitivity may be related to apoptosis escaping and (or) autophagy induction in cells. Apoptosis is the type I programmed cell death [9]. Its correlation with radiosensitivity has been intensively studied by numerous scholars. However, autophagy is the type II programmed cell death [10, 11]. Its intricate relationships with radiosensitivity, hypoxia and HIF-1α have not been clearly elaborated [9, 11]. The present study aimed to evaluate the effect of HIF-1α on inflammation in spinal cord injury (SCI) to uncover the molecular mechanisms of anti-inflammation.

## Materials and methods

### SCI model

Male Sprague–Dawley rats (6 weeks, 220–250 g) were purchased from Animal Experimental Center of Zhejiang University. All rats were housed at 22–23 °C, 55–60% humidness, 8:00–20:00 light/ dark cycle, and received standard rodent chow and access to water ad libitum. This study was approved by Ethical Committee of The First Affiliated Hospital of Zhejiang University.

Rats were fixed in the prone position and anesthetized by 35 mg/kg of pentobarbital. Skin was cut to 2–3 cm middle incision in the back, and vertebral T7–T9 were exposed. After stabilizing vertebral T7 and T9, a laminectomy was performed at the thoracic level T8. A syringe needle was used to induce the injury, which was released from a height of 12.5 mm above the surface of the cord, inflicting a moderate contusion. Hemostatic suture was performed layer by layer, and alcohol was then applied for disinfection.

### BBB score, the water content of spinal cord and HE staining

After 24 h of induction SCI, BBB test was performed as locomotor rating scale of 0 (no observable hind-limb movements) to 21 (normal locomotion) [12]. After 24 h, rat was intraperitoneally anesthetized with 35 mg/kg pentobarbital sodium and sacrificed using decollation. Spinal cord tissue samples were collected and cut into two parts. One part tissue was weight as damp weight. Then, it was dried at 80 °C and weighed as dry weight. The water content of spinal cord was calculated (damp weight − dry weight) /damp weight × 100%.

Next, part tissue fixed with 4% paraformaldehyde for 24 h, dehydrated, and embedded in OCT compound. Tissues were cut in the sagittal or axial plane into 10-μm sections. Thick sections were stained with hematoxylineosin (H&E) for 15 min. Tissue sample sections were examined by a light microscope (Leica DFC280, UK).

### Gene expression profiling

Total RNA was amplified into Affymetrix HG-U133 Plus 2.0 GeneChip arrays (Affymetrix, Santa Clara, CA, USA). Data were analyzed through Database for Annotation, Visualization, and Integrated Discovery (DAVID Database), and QIAGEN’s Ingenuity Pathway Analysis (IPA, QIAGEN, Redwood City, USA).

*Real-time PCR analysis.* Total RNA extracted from tissue samples and cell samples with Trizol (Invitrogen USA) and converted into cDNA by using M-MLV reverse transcriptase and cDNA Synthesis Kit (Invitrogen USA). Real-time PCR was conducted by using iCycler RealTime PCR Detection System (Bio-Rad Laboratories, Hercules, CA, USA) with a SYBR ExScript RT-PCR kit (Takara Biotechnology Co., Ltd., Dalian, China). The thermal cycling conditions were as follows: 95.0 °C for 10 min, 40 cycles of 95.0 °C for 15 s, 60.0 °C for 30 s and 72.0 °C for 5 min. The relative expression level was presented as 2^−ΔΔCt^ [13].

*ELISA kits.* Tissue samples and cell samples were collected, and proteins were extracted by RIPA lysis buffer. 10 μg protein was used to analyze TNF-α, IL-1β, IL-6 and IL-18 levels using ELISA KITS.

### Cell culture and transfection

PC12 cell was incubated using the Dulbecco’s Modifed Eagle Medium (DMEM) containing 10% fetal bovine serum (FBS) at an incubator with 5% CO_2_ at 37 °C. HIF-1α plasmid, miR-143, anti- miR-143 and negative mimics were transfected into cell using Lipofectamine 3000 (Thermo Fisher Scientific, Waltham, MA, USA). After 48 h of transfection, Neuro-2a cell was induced with 200 ng/mL of LPS for 4 h. Next, after 4 h of transfection, Neuro-2a cell was treated with LC3 inhibitor (3-Methyladenine, 5 μM) or autophagy agonist (1 μM resveratrol) for 4 h, and Neuro-2a cell was induced with 100 ng/mL of LPS for 4 h.

### Western blot analysis

Cells were harvested and proteins were extracted by RIPA lysis buffer. Proteins were separated on 10% polyacrylamide gels and transferred to polyvinylidene difluoride (PVDF) membranes. The membranes were then blocked with 5% non-fat milk in Tris-buffered saline containing Tween-20 (TBST). at room temperature for 1 h and incubated with anti-HIF-1α, anti-p65, anti-LC3, anti-p62, anti-GAPDH (Cell signaling Technology, Beverly, MA, USA) at 4 °C overnight. Membranes were subsequently incubated with goat anti-rabbit peroxidase-conjugated secondary antibodies (Cell signaling Technology, Beverly, MA, USA) at 37 °C for 1 h. Membranes was detected using an enhanced chemiluminescence kit and Image Lab 3.0 (Bio-Rad Laboratories, Inc.).

### Immunohistochemistry

Cell was washed with PBS and fixed with 4% paraformaldehyde for 15 min. Cell was washed with PBS and blocked with 5%-BSA supplemented with 0.25%-Tris-X100 in PBS for 1 h at room temperature. Cell was washed with PBS and incubated with anti-HIF-1α and anti-LC3 at 4 °C overnight. Cell was washed with PBS and incubated with 555- or 488- goat anti-rabbit peroxidase-conjugated secondary antibodies (1:100) room temperature for 1 h. Cell was washed with PBS and incubated with DAPI assay for 15 min at darkness.

### Statistical analysis

All data are represented as mean ± SD for three independent experiments. Student’s t-tests or one-way analysis of variance (ANOVA) and Tukey’s post test were used for all comparisons involving continuous dependent variables. Results were considered statistically significant when the p-value was < 0.05.

## Results

### HIF-1α expression levels in SCI model rats

**Fig. 1:**
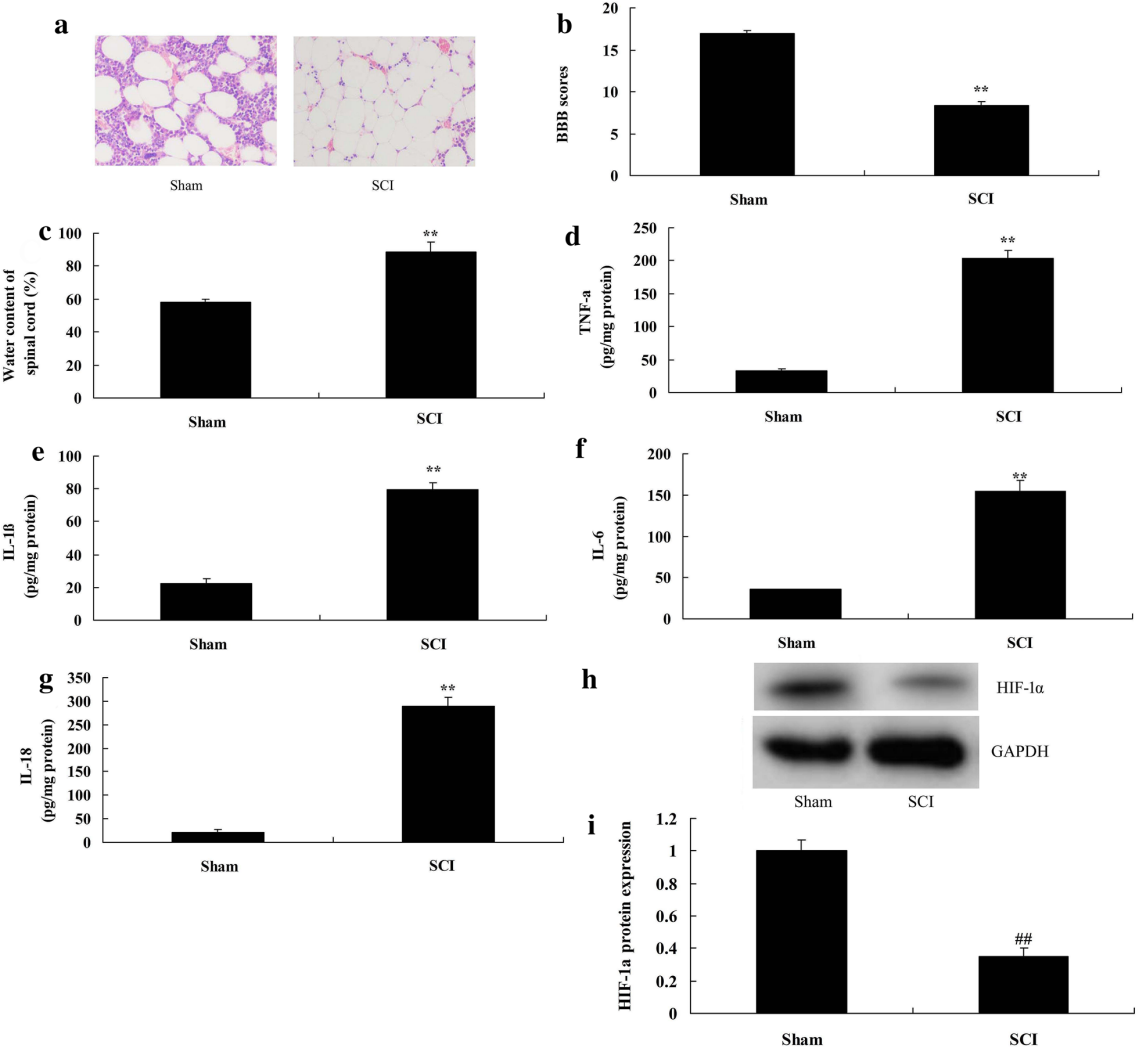
HIF-1α Expression Levels in SCI model rats. HE staining (**a**), BBB score (**b**), water content of spinal cord (**c**), TNF-α (**d**), IL-1β (**e**), IL-6 (**f**) and IL-18 (**g**), HIF-1α protein expression (**h**) and statistical analysis (**i**). *Sham* sham control group, *SCI* SCI model group. **p < 0.01 compared with sham control group

To evaluate the changes of HIF-1α expression in SCI, we analyzed HIF-1α expression using QPCR. Firstly, by HE staining, spinal cord death was observed in SCI model group (Fig. 1a). BBB score was significantly reduced and water content of spinal cord was significantly increased in SCI model group compared with sham control group (*P* < 0.05) (Fig. 1b, c). TNF-α, IL-1β, IL-6 and IL-18 levels of SCI model group were significantly increased in SCI model group compared with sham control group (*P* < 0.05) (Fig. 1d–g). As showed in Fig. 1h, i, HIF-1α protein expression was significantly down-regulated in SCI model rats compared with sham control group (*P* < 0.05).

**Fig. 2:**
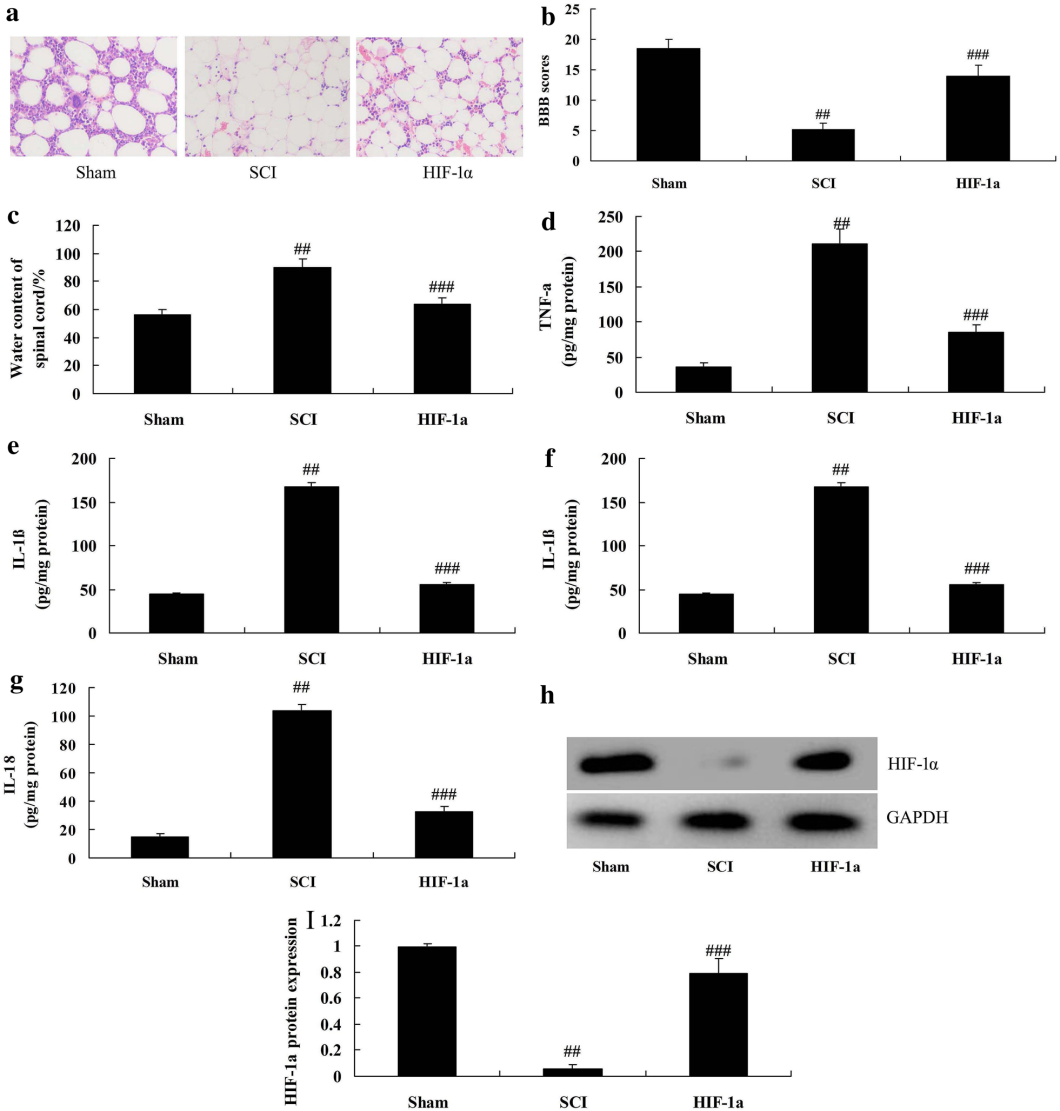
Circ 0001723 regulated inflammation in vitro model of SCI. HE staining (**a**), BBB score (**b**), water content of spinal cord (**c**), TNF-α (**d**), IL-1β (**e**), IL-6 (**f**) and IL-18 (**g**), HIF-1α protein expression (**h**) and statistical analysis (**i**). *Sham* sham control group, *SCI* SCI model group. **p < 0.01 compared with sham control group

In rat model, HIF-1α recombination protein was injected into SCI rat. It was found that spinal cord death was reduced, while BBB score was significantly increased, and water content of spinal cord was significantly decreased in SCI model with HIF-1α treatment group compared with SCI model group (*P* < 0.05) (Fig. 2a–g). HIF-1α protein expression was significantly up-regulated in SCI model rats with HIF-1α treatment compared with SCI model group significantly (Fig. 2h, i).

### Circ 0001723 regulated inflammation in vitro model of SCI

**Fig. 3:**
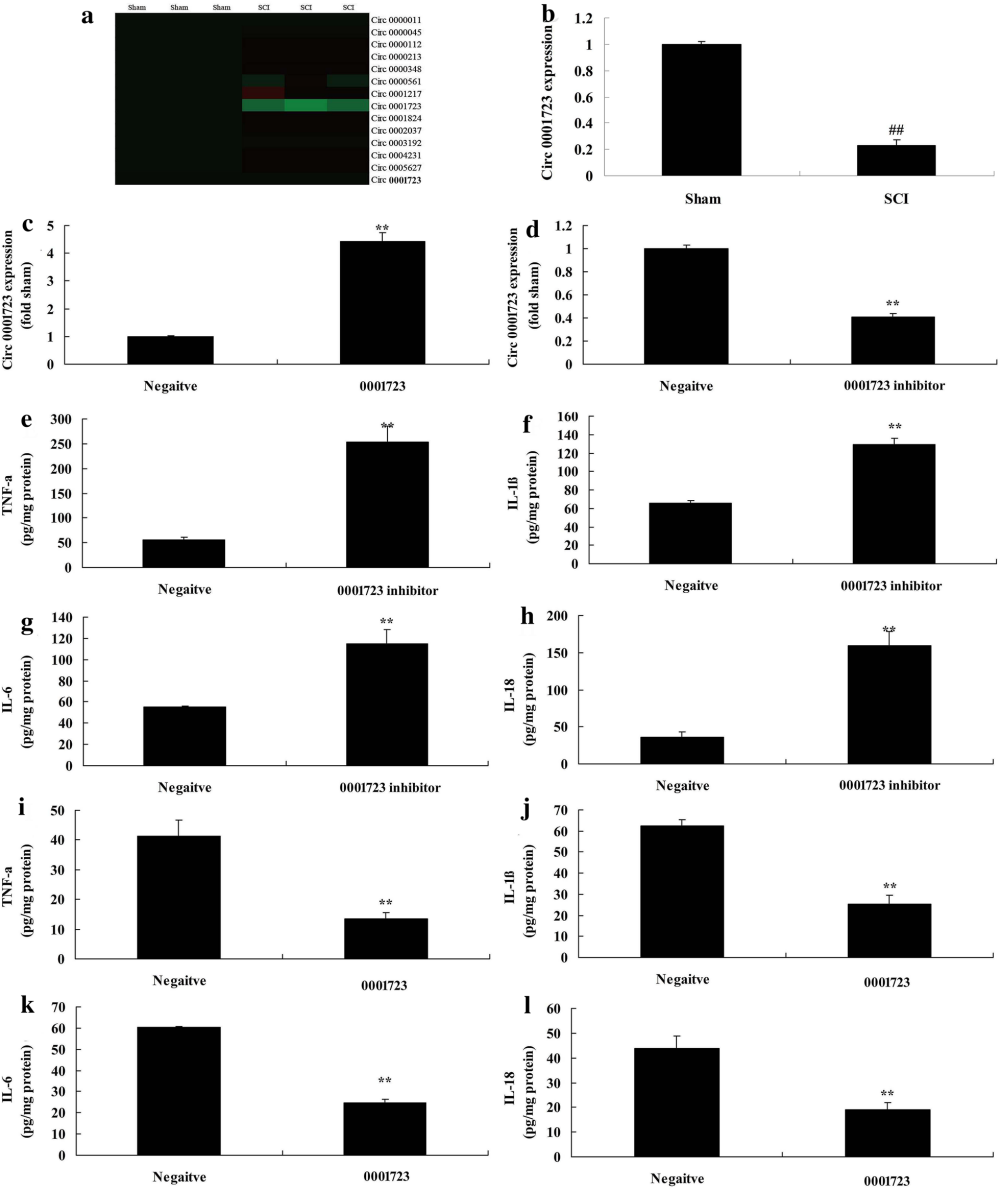
Circ 0001723 expression in vitro model of SCI. Gene chip and QPCR for Circ 0001723 expression (**a** and **b**) in vitro model of SCI; Circ 0001723 expression (**c** and **d**), TNF-α (**e**), IL-1β (**f**), IL-6 (**g**) and IL-18 (**h**) by down-regulation of Circ 0001723; TNF-α (**i**), IL-1β (**j**), IL-6 (**k**) and IL-18 (**l**) by over-expression of Circ 0001723. Negative, negative control group; 0001723, over-expression of Circ 0001723 group; 0001723 inhibitor, down-regulation of Circ 0001723 group. **p < 0.01 compared with negative control group

Circ 0001723 expression was significantly reduced in rat model of SCI compared with sham group (*P* < 0.05) (Fig. 3a, b). To investigate the role of Circ 0001723 in inflammation vitro model of SCI, Circ 0001723 or si-Circ 0001723 mimics were used to increase or decrease the expression of Circ 0001723 in vitro model. As showed in Fig. 3c, d, Circ 0001723 expression was significantly increased by Circ 0001723 mimics, and Circ 0001723 expression was significantly decreased by Circ 0001723 mimics compared with negative control group (*P* < 0.05). Down-regulation of Circ 0001723 significantly increased TNF-α, IL-1β, IL-6 and IL-18 levels in vitro model of SCI compared with negative control group (*P* < 0.05) (Fig. 3e–h). Over-expression of Circ 0001723 significantly decreased TNF-α, IL-1β, IL-6 and IL-18 levels in vitro model of SCI compared with negative control group (*P* < 0.05) (Fig. 3i–l).

### Circ 0001723 regulated inflammation in vitro model of SCI by HIF-1α

**Fig. 4:**
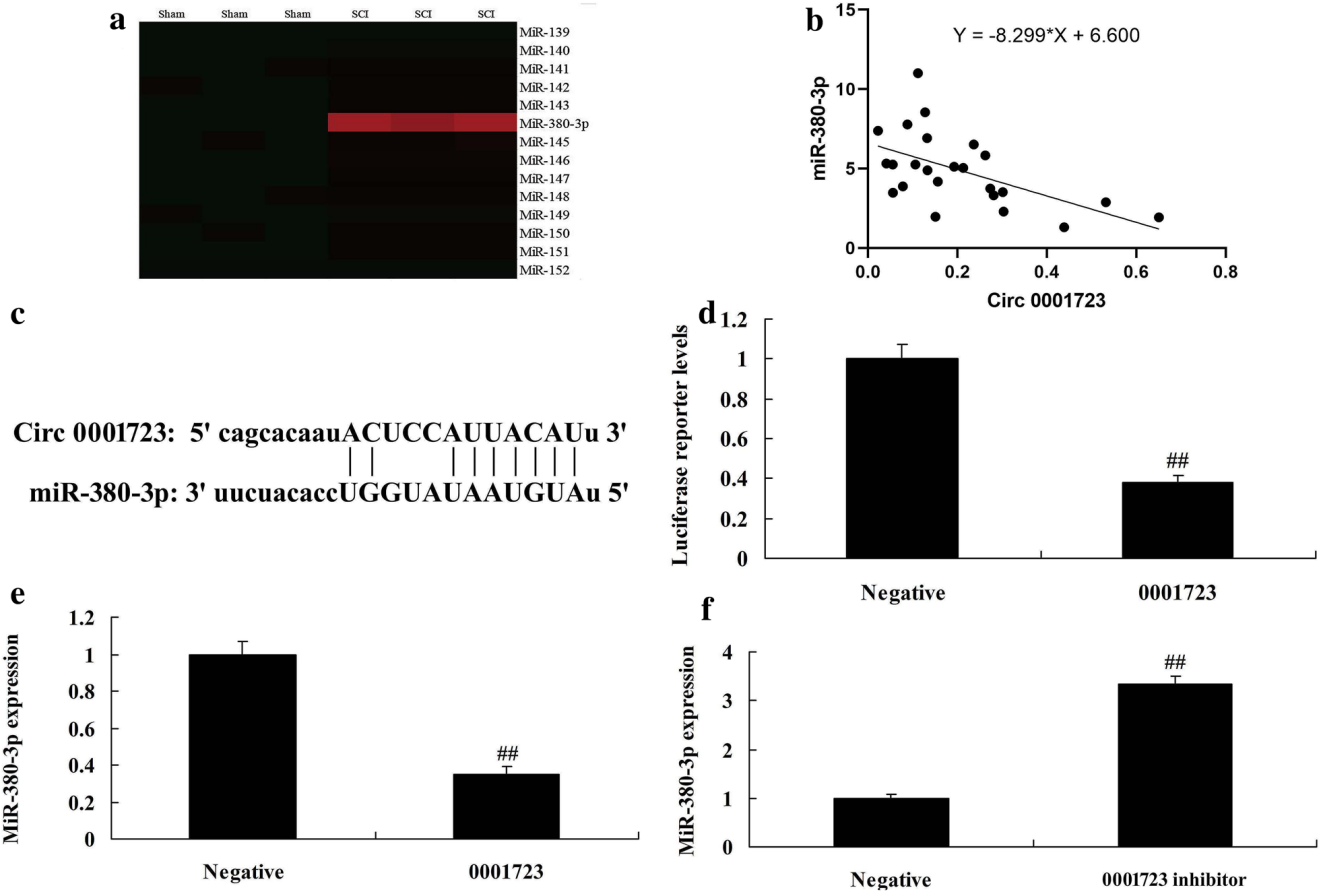
Circ 0001723 regulated inflammation in vitro model of SCI by miR-380-3p. Heat map for miR-380-3p (**a**), there was a negative correlation between Circ 0001723 and miR-380-3p (**b**), Circ 0001723 target miR-380-3p mRNA (**c**), Luciferase reporter activity (**d**), miR-380-3p expression by over-expression of Circ 0001723 group (**e**) or down-regulation of Circ 0001723 (**f**). Negative, negative control group; 0001723, over-expression of Circ 0001723 group; 0001723 inhibitor, down-regulation of Circ 0001723 group. **p < 0.01 compared with negative control group

**Fig. 5:**
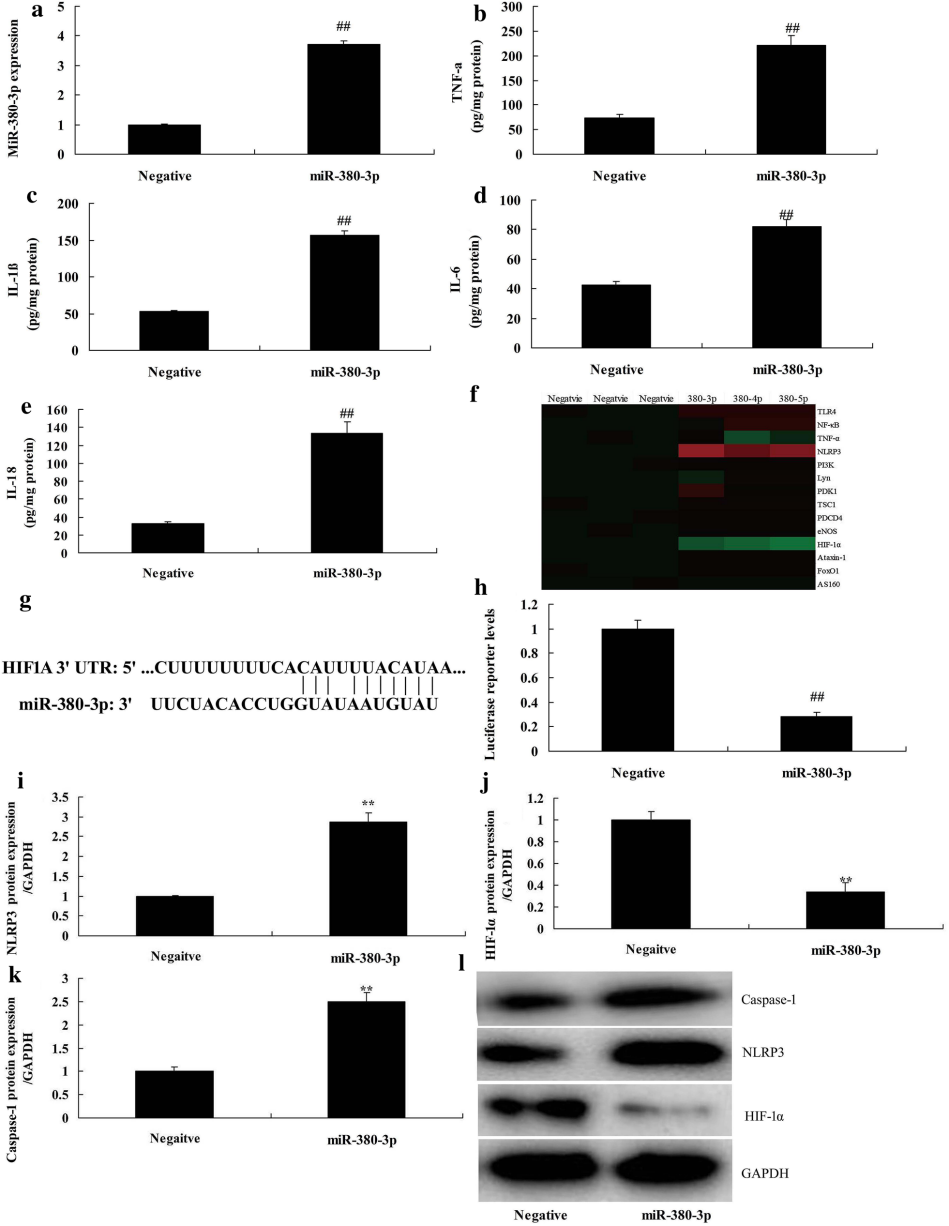
MiR-380-3p regulated HIF-1α/NLRP3 in vitro model of SCI. MiR-380-3p expression (**a**), TNF-α (**b**), IL-1β (**c**), IL-6 (**d**) and IL-18 (**e**), Heat map for HIF-1α/NLRP3 (**f**), miR-380-3p target HIF-1α mRNA (**g**), Luciferase reporter activity (**h**), HIF-1α, NLRP3 and Caspase-1 protein expression by western blot analysis (**i**–**k**) and statistical analysis (**l**). Negative, negative control group; miR-380-3p, over-expression of miR-380-3p group. **p < 0.01 compared with negative control group

**Fig. 6:**
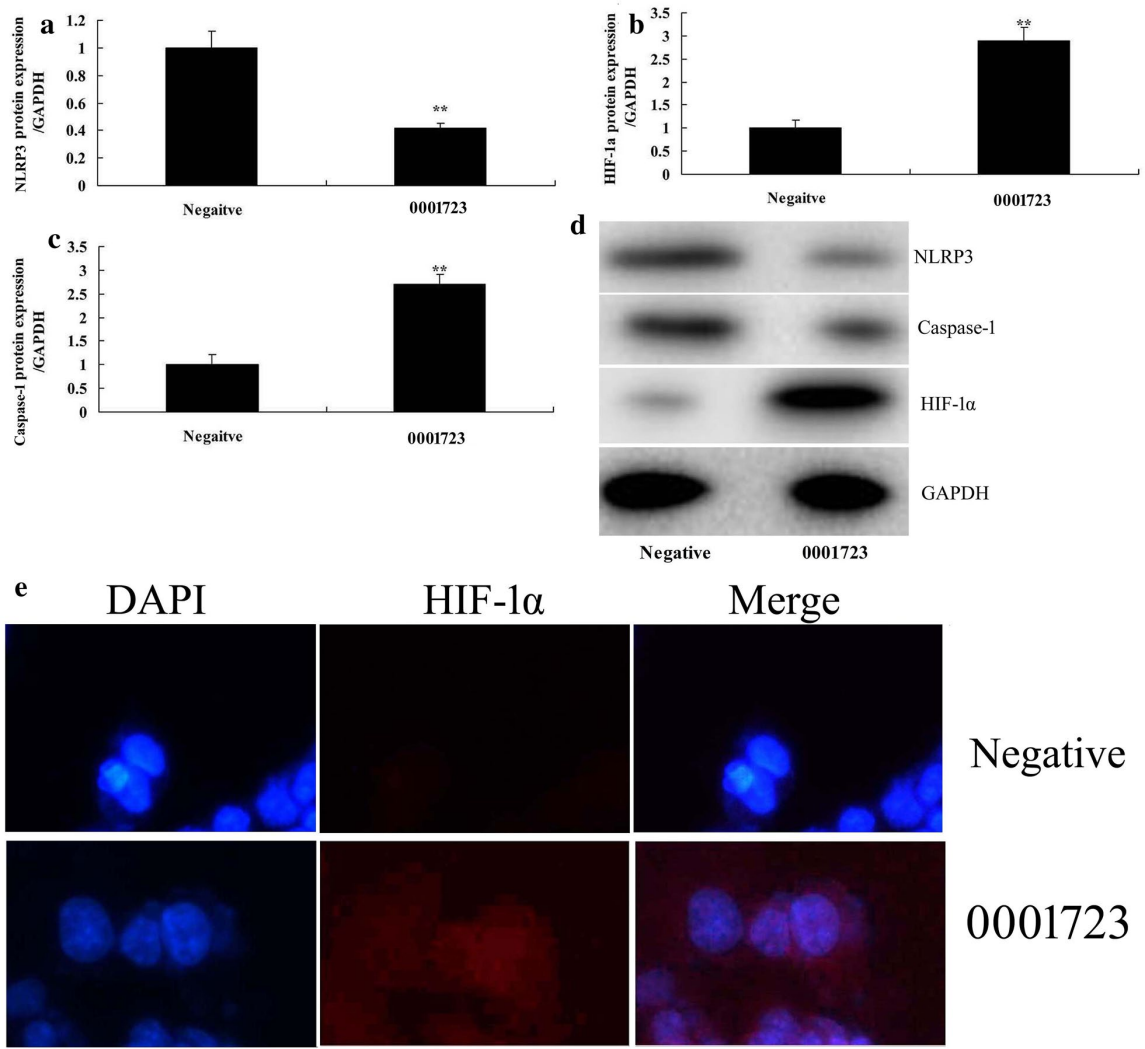
Circ 0001723 regulated HIF-1α/NLRP3 in vitro model of SCI by miR-380-3p. HIF-1α, NLRP3 and Caspase-1 protein expression by western blot analysis (**a**–**c**) and statistical analysis (**d**), HIF-1α expression (**e**). Negative, negative control group; 0001723, over-expression of Circ 0001723 group. **p < 0.01 compared with negative control group

We further investigated the function of Circ 0001723 on regulating inflammation in vitro model of SCI by mRNA. Gene chip showed that miR-380-3p expression was significantly increased in rat model of SCI, compared with sham group (*P* < 0.05) (Fig. 4a). There was a negative correlation between Circ 0001723 and miR-380-3p (Fig. 4b). Circ 0001723 targets 3′-UTR of miR-380-3p mRNA and luciferase reporter levels were significantly reduced in Circ 0001723 group compared with negative group (Fig. 4c, d). Circ 0001723 reduced miR-380-3p expression, and down-regulation of Circ 0001723 increased miR-380-3p expression in vitro model compared with negative group (*P* < 0.05) (Fig. 4e, f). Then, miR-380-3p mimics significantly increased miR-380-3p expression in vitro model compared with negative group (*P* < 0.05) (Fig. 5a). Over-expression of miR-380-3p significantly increased TNF-α, IL-1β, IL-6 and IL-18 levels in vitro model compared with negative group (*P* < 0.05) (Fig. 5b–e). These results of gene chip showed that miR-380-3p induced NLRP3 expression and reduced HIF-1α expression in vitro model compared with negative group (Fig. 5f). MiR-380-3p targets 3′-UTR of HIF-1α mRNA and luciferase reporter levels were significantly reduced in miR-380-3p group compared with negative group (*P* < 0.05) (Fig. 5g, h). Over-expression of miR-380-3p significantly induced NLRP3 and caspase-1 protein expression, and suppressed HIF-1α protein expression in vitro model compared with negative group (*P* < 0.05) (Fig. 5i–l). Over-expression of Circ 0001723 significantly suppressed NLRP3 and caspase-1 protein expression, and induced HIF-1α protein expression in vitro model compared with negative group (*P* < 0.05) (Fig. 6a–d). Over-expression of Circ 0001723 significantly induced HIF-1α protein expression in vitro model compared with negative group (*P* < 0.05) (Fig. 6e).

### miR-380-3p reduced the effects of HIF-1α/NLRP3 on inflammation by Circ 0001723

**Fig. 7:**
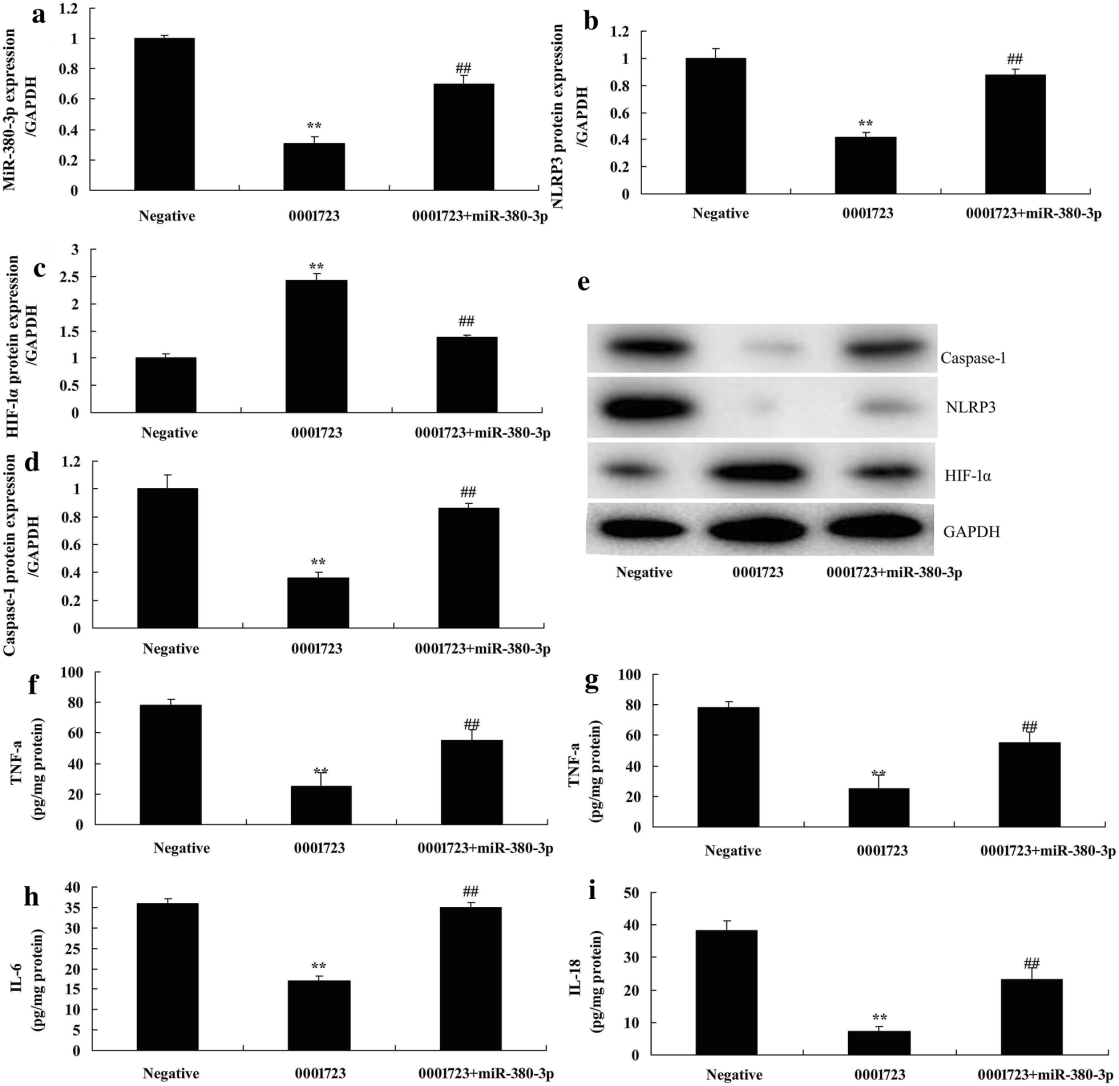
MiR-380-3p reduced the effects of HIF-1α/NLRP3 on inflammation by Circ 0001723. MiR-380-3p expression (**a**), HIF-1α, NLRP3 and Caspase-1 protein expression by western blot analysis (**b**–**d**) and statistical analysis (**e**), TNF-α (**f**), IL-1β (**g**), IL-6 (**h**) and IL-18 (**i**). Negative, negative control group; 0001723, over-expression of Circ 0001723 group; 0001723 + miR-380-3p, over-expression of Circ 0001723 and miR-380-3p group **p < 0.01 compared with negative control group, ^##^p < 0.01 compared with over-expression of Circ 0001723 group

To explore the mechanism of HIF-1α on inflammation in SCI, miR-380-3p mimics increased the miR-380-3p expression in vitro model of SCI following Circ 0001723, compared with Circ 0001723 group (Fig. 7a). Over-expression of miR-380-3p significantly induced NLRP3 and caspase-1 protein expression, and suppressed HIF-1α protein expression in vitro model following Circ 0001723 compared with Circ 0001723 group (*P* < 0.05) (Fig. 7b–e). Over-expression of miR-380-3p significantly reduced the effect of Circ 0001723 on induction of TNF-α, IL-1β, IL-6 and IL-18 levels in vitro model compared with Circ 0001723 group (*P* < 0.05) (Fig. 7f–i).

### The activation of HIF-1α reduced the pro-inflammation effects of Circ 0001723 down-regulation on inflammation in vitro model of SCI

**Fig. 8:**
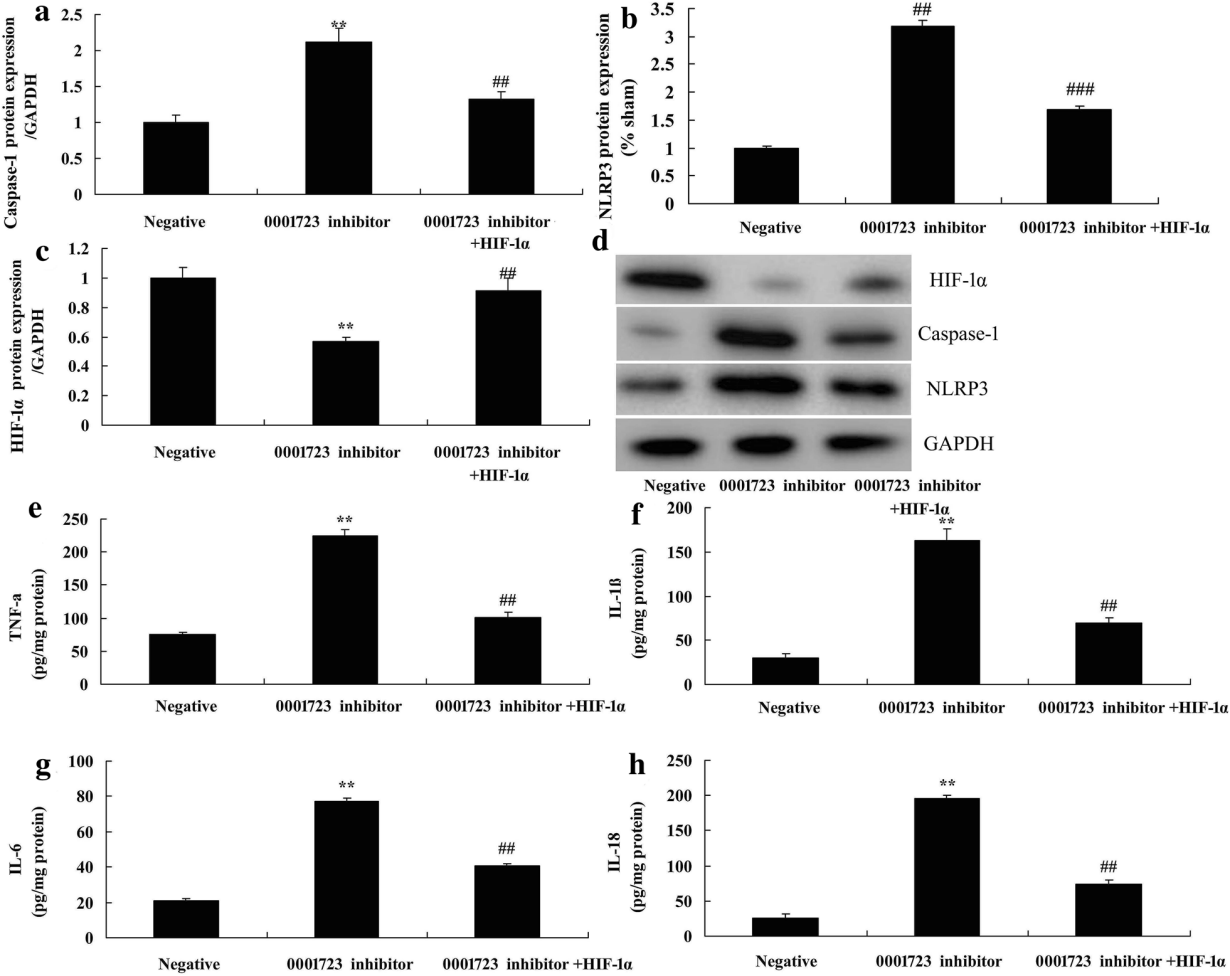
The activation of HIF-1α reduced the pro-inflammation effects of Circ 0001723 down-regulation on inflammation in vitro model of SCI. HIF-1α, NLRP3 and Caspase-1 protein expression by western blot analysis (**a**–**c**) and statistical analysis (**D**), TNF-α (**e**), IL-1β (**f**), IL-6 (**g**) and IL-18 (**h**). Negative, negative control group; 0001723 inhibitor, down-regulation of Circ 0001723 group; 0001723 inhibitor + HIF-1α, down-regulation of Circ 0001723 and over-expression of HIF-1α group. **p < 0.01 compared with negative control group, ^##^sp < 0.01 compared with down-regulation of Circ 0001723 group

We explored the role of HIF-1α in the pro-inflammation effects of Circ 0001723 down-regulation on inflammation in vitro model of SCI. HIF-1α plasmid significantly induced HIF-1α protein expression and reduced NLRP3 and caspase-1 protein expression in vitro model of SCI by Circ 0001723 down-regulation compared with Circ 0001723 down-regulation group (*P* < 0.05) (Fig. 8a–d). The activation of HIF-1α significantly reduced TNF-α, IL-1β, IL-6 and IL-18 levels in vitro model of SCI by Circ 0001723 down-regulation compared with Circ 0001723 down-regulation group (*P* < 0.05) (Fig. 8e–h).

### The inactivation of NLRP3 reduced the anti-inflammation effects of anti-Circ 0001723 on inflammation in vitro model of SCI

**Fig. 9:**
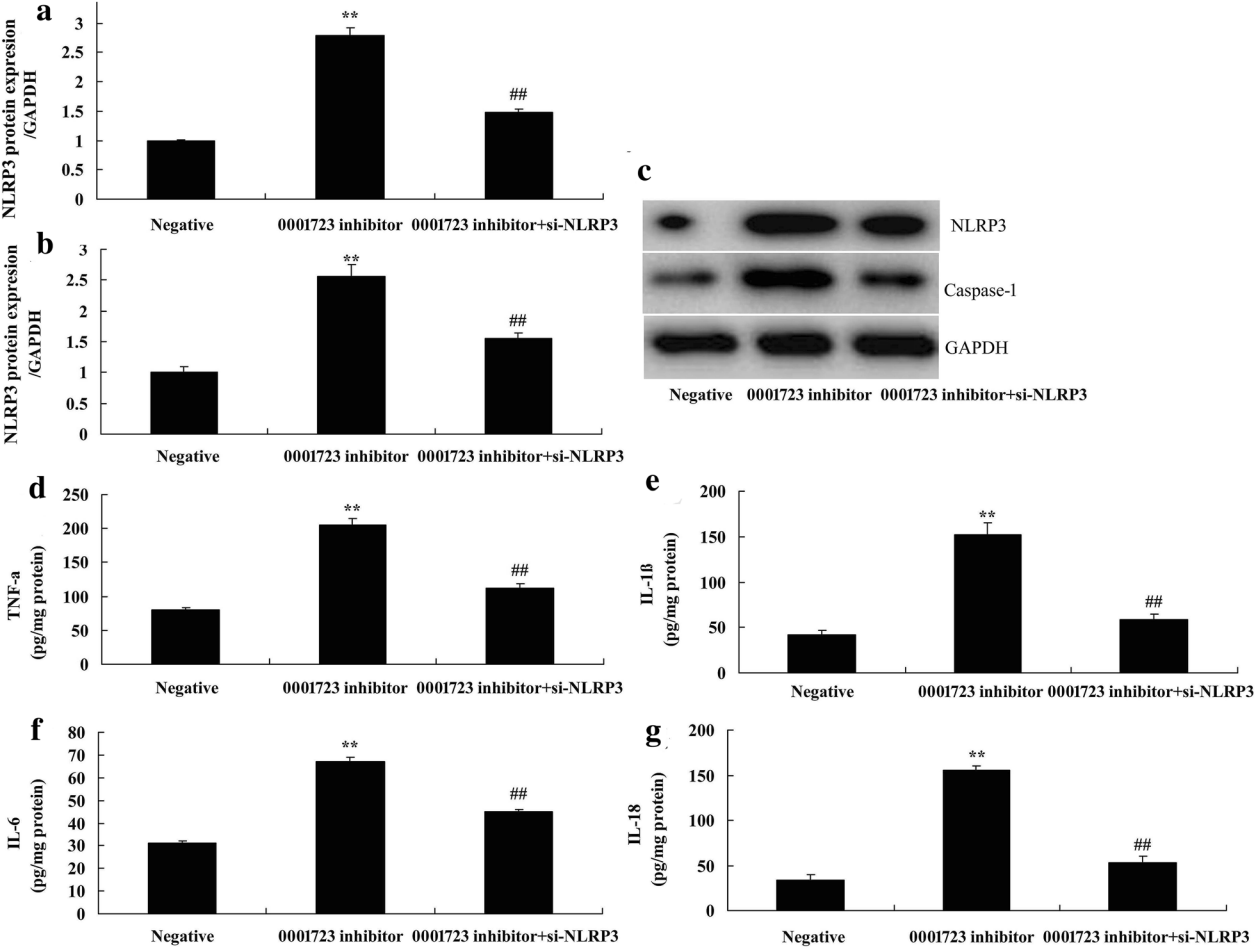
The inactivation of NLRP3 reduced the anti-inflammation effects of anti-Circ 0001723 on inflammation in vitro model of SCI. NLRP3 and Caspase-1 protein expression by western blot analysis (**a** and **b**) and statistical analysis (**c**), TNF-α (**d**), IL-1β (**e**), IL-6 (**f**) and IL-18 (**g**). Negative, negative control group; 0001723 inhibitor, down-regulation of Circ 0001723 group; 0001723 inhibitor + si-NLRP3, down-regulation of Circ 0001723 and NLRP3 group. **p < 0.01 compared with negative control group, ^##^p < 0.01 compared with down-regulation of Circ 0001723 group.

We further explored the role of NLRP3 in the anti-inflammation effects of Circ 0001723 on inflammation in vitro model of SCI. As showed in Fig. 7a–e, si-NLRP3 suppressed NLRP3 and caspase-1 *expression *in vitro model of SCI by Circ 0001723 down-regulation compared with Circ 0001723 down-regulation group (Fig. 9a–c) (*P* < 0.05). The activation of LC3 significantly reduced TNF-α, IL-1β, IL-6 and IL-18 levels in vitro model of SCI by Circ 0001723 down-regulation compared with Circ 0001723 down-regulation group (*P* < 0.05) (Fig. 9d–g).

## Discussion

SCI is one of the common severe diseases in Spine Surgery. It is associated with extremely high mortality and disability. Typically, its treatment remains a worldwide challenge. Autophagy is an important defense and protection mechanism of the body [8]. The body can remove the damaged and denatured proteins as well as organelles with function loss through autophagy [8]. Finally, it can realize cell recycle and re-utilization [14]. On the other hand, the excessive induction of autophagy will induce another programmed cell death different from apoptosis, which is the autophagic cell death. In recent years, autophagy has become the hotspot in SCI research [15]. But the role of autophagy activation in SCI remains controversial [16]. Therefore, this paper aimed to explore the relationships of post-SCI autophagy activation, autophagic cell death and autophagy with apoptosis, molecular metabolism promotion of autophagy, and the two-sideness of autophagy [16]. At present, we found that Circ 0001723 and HIF-1α expression was reduced and miR-380-3p expression was increased in SCI model. Cosin-Roger et al. showed that Hypoxia ameliorates intestinal inflammation through NLRP3 downregulation and autophagy activation [17].

Inflammatory chemokine and cytokine can supplement and activate the immune cells and central nervous cells [1]. They have play key roles in promoting and maintaining inflammatory response [18]. In any tissue, inflammatory response has its own limitation and can degrade at appropriate timing [18]. The degradation program includes cytokine transformation from pro-inflammatory to anti-inflammation [19]. Besides, it also includes the transformation of arachidonic acid from pro-inflammatory factor to the lipid medium promoting inflammation degradation [19]. The adverse effect of inflammatory response will expand if it can not end in an appropriate way in the injury region [19]. The central nervous system has limited axonal regeneration and damaged neuron supplementation [19]. Therefore, the adverse effect of inflammation is more obvious than that on other tissues. Typically, it can induce the apoptosis of neuron and oligodendrocyte and scar formation [19]. In this study, we found that HIF-1α prevented SCI and inflammation in SCI model.

Bioinformatics analysis suggests that, the potential target genes of the changed miRNA after SCI include those encoding the components during inflammation, oxidative stress and apoptosis [20]. These processes are considered to play important roles in the pathogenesis of SCI [20]. These findings indicate that, abnormal miRNA expression may play a role in the pathogenesis of SCI [21]. Besides, they may serve as the potential targets of the therapeutic intervention after SCI [21]. Our study showed that Circ 0001723 targets 3′-UTR of miR-380-3p mRNA and MiR-380-3p targets 3′-UTR of HIF-1α mRNA. Assalin et al. reported that miR-380-3p expression was increased in the gestational protein-restricted left ventricle [22].

Research indicates that, HIF-1α expression is increased under hypoxia and ischemia/reperfusion status [23]. Intervening HIF-1α expression can suppress the activation of autophagy [24]. This reveals that autophagy activation induced by hypoxia and ischemia/reperfusion is closely correlated with high HIF-1α expression [24]. More importantly, intermittent hypoxia can also induce the increased HIF-1α expression [9]. Therefore, HIF-1α may participate in the intermittent hypoxia-induced autophagy activation [25]. Besides, HIF-1α can induce the over-expression of transcription factor p62. Subsequently, it can release p62 from its binding with Bcl-2 protein, and the activated p62 can take part in forming autophagy [25]. In our study, we have found that HIF-1α was reduced in SCI model rats, which was contrary to the previous study [26]. Although HIF-1α could be obviously increased under hypoxic conditions, it was easily decreased under normoxic conditions [27]. We have only measured the expression of HIF-1α at 24 h after SCI, which might cause the difference. It was reported that HIF-1α was significantly increased after 24 h [28]. This is also the limitation of our study. Further study with multiple time points was needed. In this experiment, it was found that MiR-380-3p reduced the effects of HIF-1α/NLRP3 on inflammation by Circ 0001723. The activation of HIF-1α or inactivation of NLRP3 reduced the pro-inflammation effects of Circ 0001723 down-regulation on inflammation in vitro model of SCI. Another limitation of this study was that Autophagy dependence was investigated by the use of inhibitor. However, 3-Methyladenine can affect other metabolic processes not related to autophagy, including several PI3Ks, while resveratrol is also an antioxidant, which could also affect other metabolic processes. Therefore, further study evaluating autophagy by determining LC3 flux was needed.

## Conclusions

**Fig. 10:**
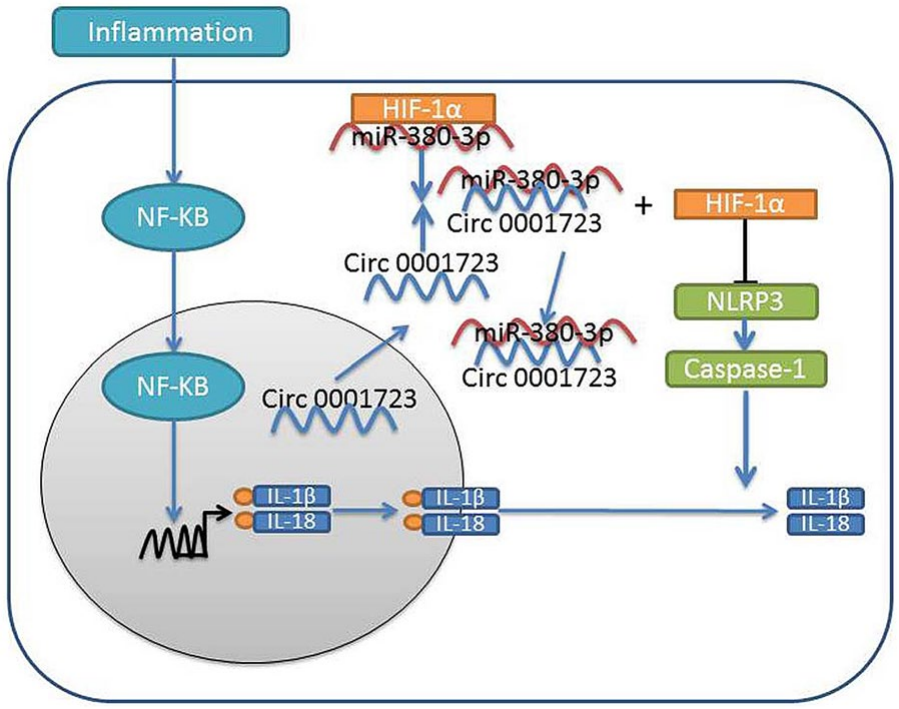
Hypoxia inducible factor-1 (HIF-1α) reduced inflammation in spinal cord injury via miR-380-3p/ NLRP3 by Circ 0001723

We showed for the first time in our study that Circ 0001723 promoted inflammation, and induced NF-κB protein expression in SCI via suppression of Autophagy by HIF-1α (Fig. 10). These results suggested the value of miR-143 as a potential new target for the treatment of SCI.

## Data Availability

The datasets used and/or analysed during the current study are available from the corresponding author on reasonable request.

## Abbreviations

SCI: Spinal cord injury
miRNA: microRNA
EAA: Excitatory amino acid
circRNAs: Circular RNAs
H&E: Hematoxylineosin
DMEM: Dulbecco’s Modifed Eagle Medium
FBS: Fetal bovine serum
PVDF: Polyvinylidene difluoride
TBST: Tris-buffered saline containing Tween-20
